# TLR4/AP-1-Targeted Anti-Inflammatory Intervention Attenuates Insulin Sensitivity and Liver Steatosis

**DOI:** 10.1155/2020/2960517

**Published:** 2020-09-16

**Authors:** Xiang Hu, Jing Zhou, Sha-sha Song, Wen Kong, Yan-Chuan Shi, Lu-Lu Chen, Tian-Shu Zeng

**Affiliations:** ^1^Department of Endocrinology, Union Hospital, Tongji Medical College, Huazhong University of Science and Technology, Wuhan, China; ^2^Hubei provincial Clinical Research Center for Diabetes and Metabolic Disorders, Wuhan, China; ^3^Department of Nutrition and Food Science, Texas A&M University, College Station, TX, USA; ^4^Department of rehabilitation medicine, Taikang Tongji (Wuhan) Hospital, Wuhan, China; ^5^Diabetes and Metabolism Division, Garvan Institute of Medical Research, St Vincent's Hospital, Sydney 2010, Australia; ^6^Faculty of Medicine, UNSW Australia, Sydney 2052, Australia

## Abstract

Insulin resistance has been shown to be the common pathogenesis of many metabolic diseases. Metainflammation is one of the important characteristics of insulin resistance. Macrophage polarization mediates the production and development of metainflammation. Toll-like receptor 4 (TLR4) mediates macrophage activity and is probably the intersection of immunity and metabolism, but the detailed mechanism is probably not fully understood. Activated protein 1 (AP1) signaling pathway is very important in macrophage activation-mediated inflammation. However, it is unclear whether AP1 signaling pathway mediates metabolic inflammation in the liver. We aimed to investigate the effects of macrophage TLR4-AP1 signaling pathway on hepatocyte metabolic inflammation, insulin sensitivity, and lipid deposition, as well as to explore the potential of TLR4-AP1 as new intervention targets of insulin resistance and liver steatosis. TLR4 and AP1 were silenced in the RAW264.7 cells by lentiviral siRNA transfection. In vivo transduction of lentivirus was administered in mice fed with high-fat diet. Insulin sensitivity and inflammation were evaluated in the treated cells or animals. Our results indicated that TLR4/AP-1 siRNA transfection alleviated high-fat diet-induced systemic and hepatic inflammation, obesity, and insulin resistance in mice. Additionally, TLR4/AP-1 siRNA transfection mitigated palmitic acid- (PA-) induced inflammation in RAW264.7 cells and metabolic abnormalities in cocultured AML hepatocytes. Herein, we propose that TLR4-AP1 signaling pathway activation plays a crucial role in high fat- or PA-induced metabolic inflammation and insulin resistance in hepatocytes. Intervention of the TLR4 expression regulates macrophage polarization and metabolic inflammation and further alleviates insulin resistance and lipid deposition in hepatocytes.

## 1. Introduction

Insulin resistance (IR) has been shown to be the common pathogenesis of many metabolic diseases, such as obesity, type 2 diabetes, cardiovascular disease, nonalcoholic fatty liver disease, and metabolic syndrome [1, 2]. It is increasingly considered that the pathogenesis of insulin resistance is complex, including inflammation, obesity, mitochondrial dysfunction, endoplasmic reticulum stress, and oxidative stress [3]. In recent years, chronic low-grade inflammation, also known as metabolic inflammation, has been identified as a major mediator of insulin resistance in obese individuals [1–4]. Studies have shown that obesity or a long-term high-fat diet leads to an increase in the levels of various inflammatory factors including TNF-*α*, IL-1, IL-6, and MCP-1 and decreases in the anti-inflammatory factors such as IL-10 and IL-4 in insulin target tissues in insulin-resistant individuals [1]. It is increasingly considered that macrophages M1-type inflammatory polarization and the activation of inflammatory pathway in major insulin target organs such as the liver and adipose tissue are particularly critical in the pathogenesis of metabolic inflammation [4].

The liver is a key metabolic organ, and insulin resistance in the liver is increasingly considered to play a crucial role in the etiology of these metabolic disorders, in which metabolic stress in the liver can induce immune activation and mediate metabolic abnormalities [5, 6]. Intrahepatic colonization of macrophages (Kupffer cells) is very important in maintaining metabolic homeostasis, and M2 type is dominant under physiological conditions, producing anti-inflammatory cytokine IL-10 [6]. In insulin resistant mice developed by high-fat diet feeding, intrahepatic macrophages are mainly characterized by M1-type polarization, which induces metabolic inflammation and mediates hepatic insulin resistance [7]. Papackova et al. reported that removal of the Kupffer cells by barium chloride prevents metabolic inflammation and insulin resistance in the liver caused by high-fat diet and reduces circulating inflammation and systemic insulin resistance [8, 9], suggesting that the Kupffer cells may play the key role in the pathogenesis in metabolic inflammation and insulin resistance. Therefore, it is very important to investigate intervention target to attenuate the Kupffer cell inflammatory activation in the prevention and treatment of metabolic inflammation and hepatocyte insulin resistance.

Recent studies indicate that toll-like receptor 4 (TLR4) is probably the intersection of immunity and metabolism. TLR4 may be the trigger of metabolite-mediated Kupffer cells activation, which plays critical role in the pathogenesis of high fat-induced inflammation and insulin resistance [2], but the detailed mechanism is probably not fully understood. Activated protein 1 (AP1) is a transcription factor that mediates the production of inflammatory cytokines. It is reported that the activation of JNK/AP1 signaling pathway is very important in the production of inflammatory factors and macrophage activation-mediated inflammation. However, it is unclear whether AP1 signaling pathway mediates metabolic inflammation in the liver. We aimed to investigate the effects of macrophage TLR4-AP1 signaling pathway on hepatocyte metabolic inflammation, insulin sensitivity, and lipid deposition, as well as to explore the potential of TLR4-AP1as new intervention targets in the prevention and treatment of metabolic inflammation and insulin resistance in the liver by alleviating the Kupffer cell inflammatory activation.

## 2. Materials and Methods

### 2.1. Animals and Diets

Experiments were performed on male C57BL/6 J mice (aged six weeks, weighing 20–23 g) as described previously [10]. All of them were housed in cages with free access to water and subjected to controlled temperature (22 ± 3°C), lighting (lights on 06 : 00–18 : 00), and relative humidity (60 ± 10%) [11]. All mice were adapted to surrounding environments for a week before the experiments and then randomly allocated to five groups, with eight mice (*n* = 8) in each group. One group was fed with normal chow (NC, 13.68%, 64.44%, and 21.88% of calories derived, respectively, from fat, carbohydrate, and protein) [12] for 16 weeks. The other four groups were fed with high-fat diet (containing in terms of calories derived 59% fat, 20% carbohydrate, and 21% protein) [13] for 16 weeks, with three groups for further intervention of lentiviral transfection and one group as control without lentiviral transfection. All the experimental procedures for the use of animals in research involved in this study were approved by the Animal Ethics Committee of the university and in accordance with the Laboratory Animal Care Guidelines of Hubei Province [11].

### 2.2. Cell Culture

RAW264.7 cells (mouse macrophage cell line) and AML12 mouse liver cells were obtained from the American Type Culture Collection. RAW264.7 cells were grown in Dulbecco's modified Eagle's medium, and AML12 cells were cultured in Dulbecco's modified Eagle's medium/Ham's F-12 (1 : 1) medium (Gibco, Carlsbad, CA, United States of America), containing 10% fetal bovine serum (Invitrogen) at 37°C in an incubator with 5% CO2 [14, 15].

### 2.3. Lentivirus Preparation and Infection

TLR4 and AP1 were silenced in the RAW264.7 cells by lentiviral siRNA transfection. Briefly, TLR4 and AP1 siRNAs were subcloned into the pcDNA6.2 vector with the enhanced green fluorescent protein (EGFP) gene and then cloned into the lentiviral vectors obtained from R&S Biotechnology Co., Ltd. (Shanghai China). All siRNA of gene *tlr4* and *ap1* were designed and synthesized by Shanghai R&S Biotechnology Co. Ltd. (Shanghai, China), and sequences were present in supplemental Table 1. The resulting plasmid was analyzed and verified by gel electrophoresis and sequencing. Virus particles were packaged in 293 T cells, and then, the virus particles were collected and tittered after 48 h transfection. The suppression efficiency was evaluated by the expression of EGFP and TLR4 and AP1 mRNAs to screen out one lentiviral packaging siRNA with highest interference efficiency for subsequent experiments. RAW264.7 cells were transfected with virus particles with TLR4 siRNA (TLR4-pLV), AP1 siRNA (AP1-pLV), or control lentivirus (Scramble-pLV), respectively [16]. Transduction efficiency was determined by measuring the green fluorescent protein. In vivo transduction of lentivirus was achieved through tail vein injections of 0.1 ml of virus particle suspension with a viral titer of 1 × 10^8^ TU/ml in mice fed with high-fat diet at 12 weeks. The injection was repeated, every two weeks for four weeks. Fourteen days after the second intravenous injection, mice were sacrificed, and the tissues were removed, weighed, fixed, or stored in liquid nitrogen for the following assays [17].

### 2.4. Glucose Tolerance Test (GTT) and an Insulin Tolerance Test (ITT)

GTT and ITT were performed on mice at 16 weeks (*n* = 4 or 5 per group) as reported previously [18]. Mice were fasted overnight for the GTT and then given an intraperitoneal injection of 2 g/kg body weight glucose at 09 : 00. Blood glucose levels in blood drawn from the tail vein were measured immediately prior to injection (time point 0) and then at 30, 60, and 120 min after injection. For the ITT, mice were fasted for 6 h and then given an intraperitoneal injection of 1 U/kg body weight insulin (Novo Nordisk) at 14 : 00. Blood glucose levels were measured immediately prior to injection (time point 0) and then at 15, 30, 60, and 90 min after injection. Blood glucose was measured using an automatic glucometer (One Touch, Lifescan) [19]. The area under the curve (AUC) was calculated for the further analysis.

### 2.5. RNA Extraction, Reverse Transcription (RT)-PCR, and Real-Time PCR

Total RNA was isolated by TRIzol reagent (Invitrogen), reverse transcribed into complementary DNA (cDNA) by MML-V reverse transcriptase (Invitrogen), and the Real-Time PCR System was used according to the manufacturer's protocol as described previously [11]. Sequences of the primers used for the real-time PCR amplification were listed in Supplemental Table 1. The relative gene expression was evaluated by normalization to the abundance of the indicated housekeeping gene expression.

### 2.6. Immunoblot Analysis

Tissue extracts were prepared, and immunoblot was performed as reported previously. Antibodies used for immunoblotting assays were obtained from Cell Signaling Technology (TLR4, Akt, p-Akt (Ser^473^), p-JNK (Ser^63^ and Ser^73^), JNK, AMPK, p-AMPK*α* (Thr^172^), mTOR, p-mTOR (Ser^2448^), and GAPDH).

### 2.7. ELISA Assay for Insulin, TNF-*α*, IL-6, IL-*β*, and IL-10

Plasma insulin levels were measured after overnight fasting by using the enzyme-linked immunosorbent assay (ELISA) kits (ALPCO) according to the manufacturer's protocol as described previously. The plasma levels of TNF-*α* and IL-6, as well as the concentrations of TNF-*α*, IL-6, IL-*β*, and IL-10 in culture supernatant, were determined by a sandwich ELISA (Beijing 4A Biotech Co., Ltd) [9] [20].

### 2.8. Determination of Serum Free Fatty Acid (FFA), Triglyceride (TG), and Total Cholesterol (TC)

Serum FFA, TG, and TC were assayed using the commercial kits from Nanjing Jiancheng Bioengineering Institute (Nanjing, China) according to the manufacturer's protocol as described previously [10].

### 2.9. Oil Red O Staining

Cells were stained with Oil Red O (Sigma) as described previously [21] and imaged using a microscope (OLYMPUS IX71, Olympus, Tokyo, Japan).

### 2.10. Statistical Analysis

Statistical analyses were performed to test the differences among the groups using 2-tailed unpaired Student *t*-test or one-way analysis of variance followed by least significant difference (LSD) *t*-test posthoc test for multiple comparisons with SPSS, version 16.0 (SPSS, Inc., Chicago, IL, USA). Bands were quantified using the Image Lab 5.0 (Bio-Rad Laboratories, Inc., Hercules, CA, USA). The results are presented as means ± S.E.M, and *p* < 0.05 was considered statistically significant.

## 3. Results

### 3.1. TLR4/AP-1 siRNA Transfection Alleviated Systemic and Hepatic Inflammation

**Figure 1:**
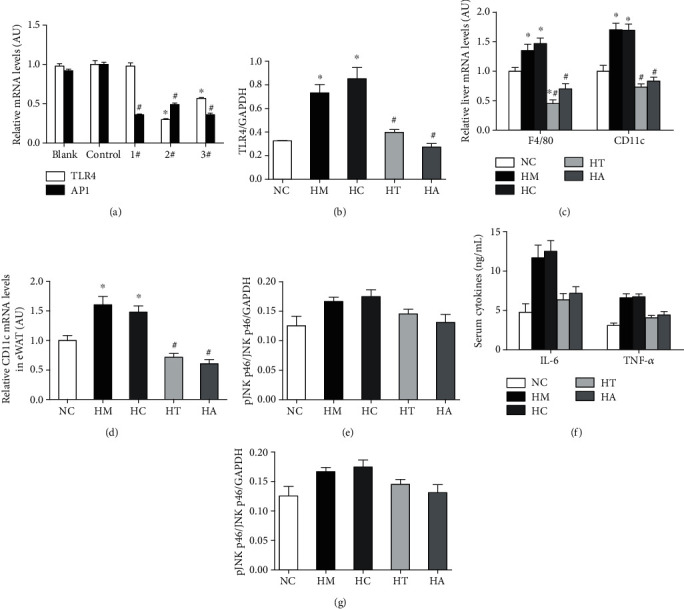
TLR4/AP-1 siRNA transfection alleviated systemic and hepatic inflammation. AP1 siRNA (1#, 2#, and 3#) and TLR4 siRNA (2# and 3#) transfection significantly down-regulated the AP1 and TLR4 mRNA expression, respectively. The decreases in the mRNA expression seemed most remarkable in 2# TLR4 siRNA and 3# AP1 siRNA transfection (a). The expression of TLR4 protein was significantly inhibited in the high-fat diet group with either TLR4 (HT) or AP1 (HA) siRNA transfection (b). The expression of F4/80 and Cd11c mRNA was upregulated in the liver of high-fat diet controls (HM) and high-fat diet controls with empty vector transfection (HC), and these changes were attenuated with either TLR4 (HT) or AP1 (HA) siRNA transfection compared with normal diet controls (NC) (c, d). No significant difference was observed in JNK phosphorylation in the liver, but the serum IL-6 and TNF-*α* levels were elevated in HM and HC, which were attenuated in HT and HA (e, f). AMPK phosphorylation was significantly decreased in the HM and HC, and it was alleviated in HT and HA (g). ∗*p* < 0.05 and ∗∗*p* < 0.01 compared with the NC group; #*p* < 0.05 and ##*p* < 0.01 compared with the HM group.

In AP1 siRNA transfection, 1#, 2#, and 3# can significantly downregulate the mRNA expression. In TLR4 siRNA transfection, both 2# and 3# siRNA transfections could significantly downregulate the mRNA expression, while 1# siRNA transfection was not obvious. Additionally, the decreases in the mRNA expression seemed most remarkable in 2# TLR4 siRNA and 3# AP1 siRNA transfection (Figure 1(a)). Thus, we used 2# TLR4 siRNA and 3# AP1 siRNA for further investigation. The expression of TLR4 was significantly inhibited in the high-fat diet group with TLR4 siRNA transfection (HT) and in the high-fat diet group with AP1 siRNA transfection (HA) to some extent (Figure 1(b)). Compared with normal diet controls (NC), the expression of F4/80 and Cd11c mRNA was upregulated in the liver of high-fat diet controls (HM) and high-fat diet controls with empty vector transfection (HC), and these changes were attenuated with TLR4 (HT) and AP1 (HA) siRNA transfection (Figures 1(c) and 1(d)). The JNK phosphorylation in the liver seemed to increase, but no significant difference was observed among these groups. However, the serum IL-6 and TNF-*α* levels were elevated in HM and HC, while these changes were attenuated with TLR4 or AP1 siRNA transfection (HT and HA) (Figures 1(e) and 1(f)). Compared with the NC group, AMPK phosphorylation was significantly decreased in the HM and HC groups, and it was alleviated in the HT and HA groups (Figure 1(g)).

### 3.2. TLR4/AP-1 siRNA Transfection Ameliorated High-Fat Diet-Induced Obesity and Insulin Resistance in Mice

**Figure 2:**
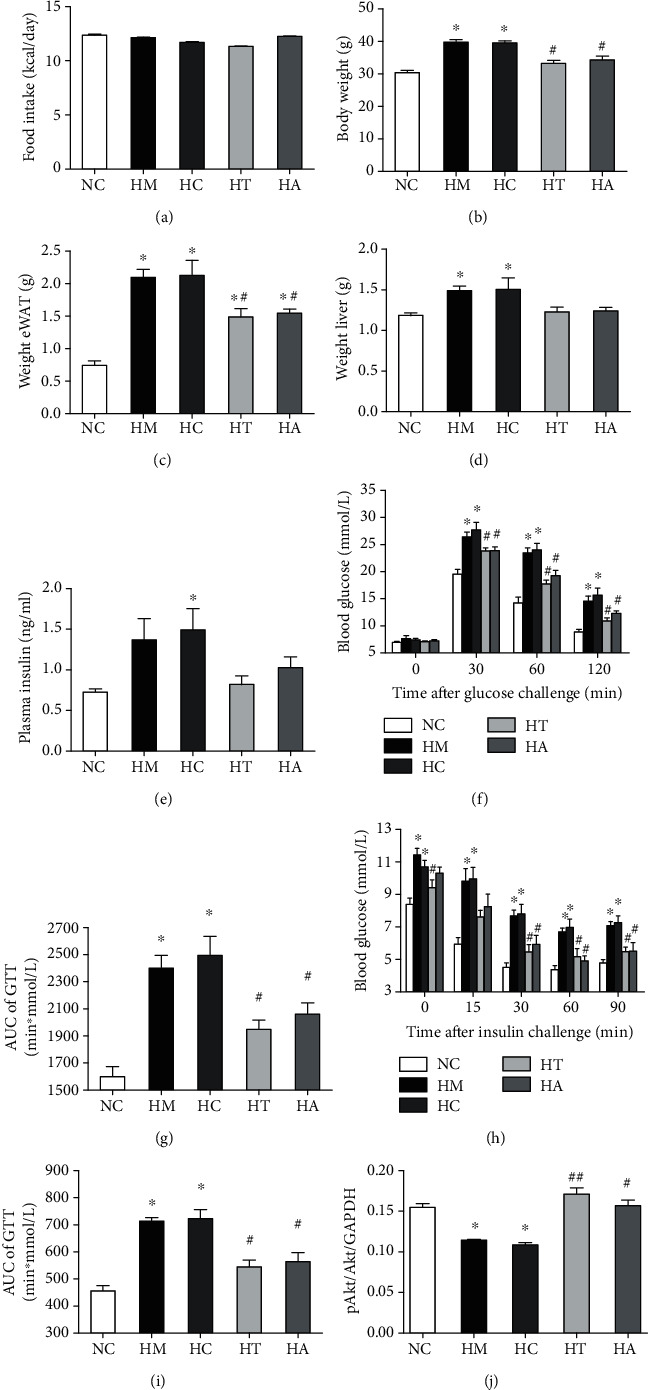
TLR4/AP-1 siRNA transfection ameliorated high-fat diet-induced obesity and insulin resistance in mice. No significant difference was observed in energy intake between NC, HM, HC, HT, and HA (a). Body weight, the weight of epididymal fat (eWAT), and wet weight of the liver were significantly increased in HM and HC compared with NC, and these changes were reversed in HT and HA (b–d). The glucose tolerance (GTT) and systemic (fasting plasma insulin level and ITT) and hepatic (Akt phosphorylation in the liver) insulin sensitivity were significantly impaired in HM and HC, and these changes were relieved in HT and HA (e–j). ∗*p* < 0.05 and ∗∗*p* < 0.01 compared with the NC group; #*p* < 0.05 and ##*p* < 0.01 compared with the HM group.

Our results showed that there was no significant difference in energy intake between NC, HM, HC, HT, and HA (Figure 2(a)). Compared with the NC group, body weight, the weight of epididymal fat (eWAT), and wet weight of the liver were significantly increased in the HM and HC groups, while TLR4 and AP1 inhibition treatments (HT and HA groups) reversed these changes (Figures 2(b)–2(d)). Additionally, the glucose tolerance (GTT) and systemic (fasting plasma insulin level and ITT) and hepatic (Akt phosphorylation in the liver) insulin sensitivity were significantly impaired in the HM and HC groups, while these changes were also relieved in the HT and HA groups (Figures 2(e)–2(k)).

### 3.3. TLR4 siRNA Transfection Mitigated PA-Induced Inflammation in RAW264.7 Cells and Metabolic Abnormalities in Cocultured AML Hepatocytes

**Figure 3:**
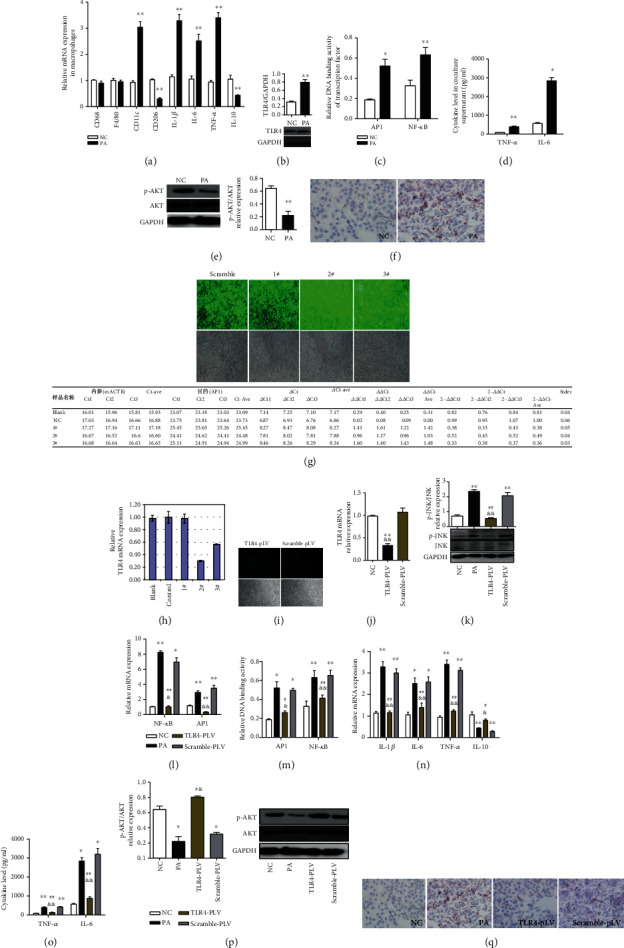
TLR4 siRNA transfection mitigated PA-induced inflammation in RAW264.7 cells and metabolic abnormalities in cocultured AML hepatocytes. The expressions of proinflammatory macrophage (M1 type) specific surface marker CD11c and proinflammatory factors (IL-1*β*, IL-6, and TNF-*α*) were significantly increased, while the expressions of anti-inflammatory macrophage (M2 type) specific surface marker CD206 and anti-inflammatory factor IL-10 were obviously decreased in palmitic acid- (PA-) treated RAW264.7 cells (a). PA treatment promoted the expression of TLR4 mRNA and protein and Ikk*β*/NF-*κ*B, activation of JNK/AP1 pathway in RAW264.7 cells, and production of proinflammatory factors (TNF-*α* and IL-6) in the supernatant (b–d). The phosphorylation of AKT was significantly decreased, and intracellular lipid accumulation was significantly increased in cocultured AML12 hepatocytes (e, f). TLR4 transfection significantly downregulated the TLR4 mRNA expression in RAW264.7 macrophages (g, j). TLR4 siRNA transfection mitigated PA-induced augmentation in JNK phosphorylation (k). TLR4 siRNA transfection could attenuate PA-induced increases in the mRNA expression and DNA binding activity of AP1 and NF-*κ*B (l, m), as well as proinflammatory factor (IL-1*β*, IL-6, and TNF-*α*) expression in RAW264.7 macrophages and TNF-*α* and IL-6 levels in the supernatant (n, o). TLR4 siRNA transfection alleviated PA-induced decreases in the mRNA expression of IL-10 (n). TLR4 siRNA transfection in RAW264.7 macrophages significantly ameliorated the decreases in AKT phosphorylation and lipid accumulation in cocultured AML12 hepatocytes (p, q). Data are presented as mean ± SEM; ∗*p* < 0.05 and ∗∗*p* < 0.01 compared with the NC group; &*p* < 0.05 and &&*p* < 0.01 compared with the Scramble-pLV group (j–n); #*p* < 0.05 and ##*p* < 0.01 compared with the PA group (k–n).

In the present study, no significant difference was observed in the expression of CD68 and F4/80 in RAW264.7 cells. However, the expressions of proinflammatory macrophage (M1 type) specific surface marker CD11c and proinflammatory factors (IL-1*β*, IL-6, and TNF-*α*) were significantly increased, while the expressions of anti-inflammatory macrophage (M2 type) specific surface marker CD206 and the anti-inflammatory factor IL-10 were obviously decreased in palmitic acid- (PA-) treated RAW264.7 cells (Figure 3(a)). PA treatment promoted the expression of TLR4 mRNA and protein accompanied by Ikk*β*/NF-*κ*B, activation of JNK/AP1 pathway in RAW264.7 cells, and production of proinflammatory factors (TNF-*α* and IL-6) in the cell culture supernatant (Figures 3(b)–3(d)). The phosphorylation level of AKT was significantly decreased, and intracellular lipid accumulation was significantly increased in cocultured AML12 hepatocytes (Figures 3(e) and 3(f)). TLR4 siRNA 2# transfection significantly downregulated the TLR4 mRNA and protein expression in RAW264.7 macrophages (Figures 3(g)–3(j)). TLR4 siRNA transfection mitigated PA-induced augmentation in JNK phosphorylation (Figure 3(k)). TLR4 siRNA transfection could attenuate PA-induced increases in the mRNA expression and DNA binding activity of AP1 and NF-*κ*B (Figures 3(l) and 3(m)), as well as proinflammatory factor (IL-1*β*, IL-6, and TNF-*α*) expression in RAW264.7 macrophages and TNF-*α* and IL-6 levels in the cell culture supernatant (Figures 3(n) and 3(o)). Meanwhile, TLR4 siRNA transfection alleviated PA-induced decreases in the mRNA expression of IL-10 (Figure 3(n)). Moreover, TLR4 siRNA transfection in RAW264.7 macrophages significantly ameliorated the decreases in AKT phosphorylation level and lipid accumulation in cocultured AML12 hepatocytes (Figures 3(p) and 3(q)).

### 3.4. AP-1 siRNA Transfection Extenuated PA-Induced Inflammation in RAW264.7 Cells and Metabolic Disorders in Cocultured AML Hepatocytes

**Figure 4:**
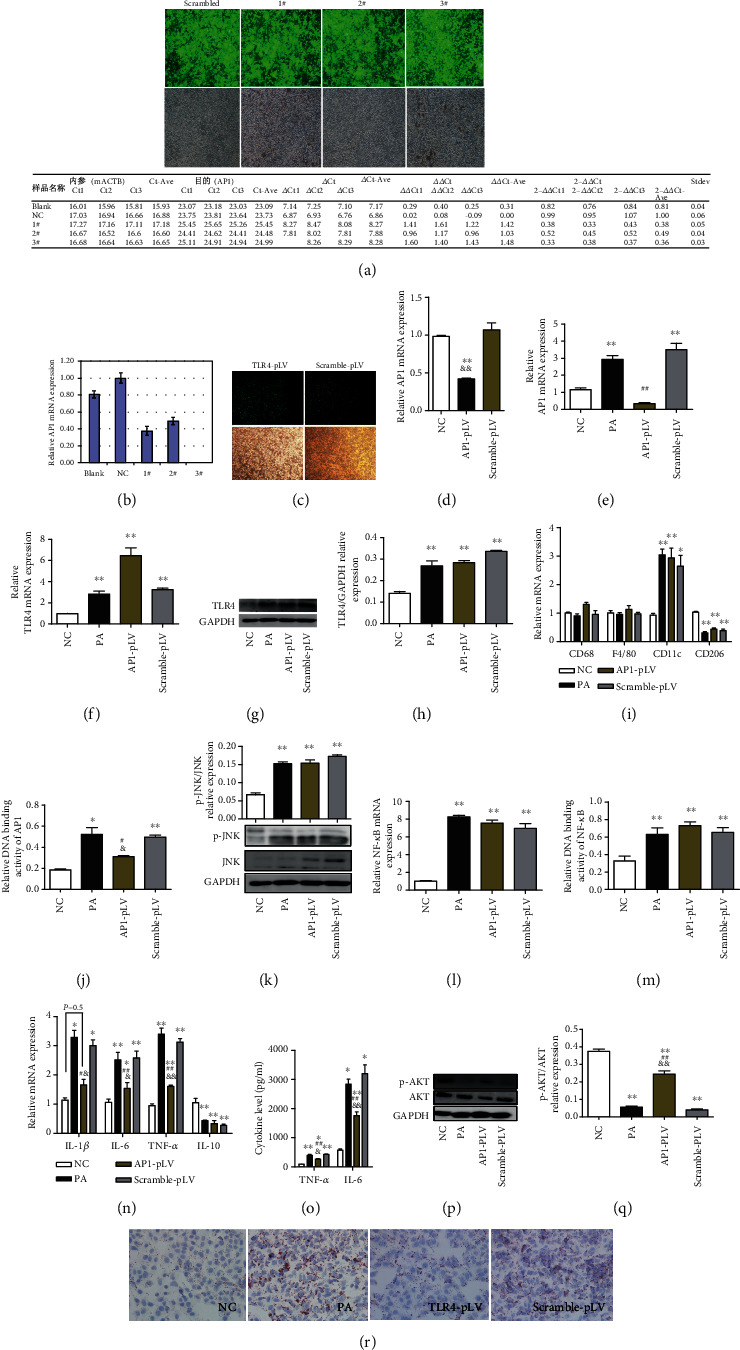
AP-1 siRNA transfection extenuated PA-induced inflammation in RAW264.7 cells and metabolic disorders in cocultured AML hepatocytes. AP1 siRNA transfection significantly diminished PA-induced elevation of the AP1 expression in RAW264.7 cells (a–e). AP1 siRNA transfection seemed to exacerbate the increases of the TLR4 expression in these cells (f–h). AP1 siRNA transfection diminished the DNA binding activity of AP1, but it seemed not be able to meliorate PA-induced increases in the CD11c mRNA expression or decreases in the CD206 mRNA expression (i, j). AP1 siRNA transfection was not able to palliate PA-induced augmentation in JNK phosphorylation (k), as well as in the mRNA expression and DNA binding activity of NF-*κ*B (l, m). AP1 siRNA transfection alleviated the increases in proinflammatory factor (IL-1*β*, IL-6, and TNF-*α*) expression in RAW264.7 macrophages and TNF-*α* and IL-6 levels in the supernatant (n, o) and attenuated PA-induced decreases in the IL-10 expression (n). AP1 siRNA transfection in RAW264.7 macrophages significantly moderated the decreases in AKT phosphorylation level and lipid accumulation in cocultured AML12 hepatocytes (p–r). Data are presented as mean ± SEM; ∗*p* < 0.05 and ∗∗*p* < 0.01 compared with the NC group; &*p* < 0.05 and &&*p* < 0.01 compared with the Scramble-pLV group (d, n, o, q); #*p* < 0.05 and ##*p* < 0.01 compared with the PA group (e, j, n, o, q).

AP1 siRNA 3# transfection significantly diminished PA-induced elevation of the AP1 mRNA expression in RAW264.7 macrophages (Figures 4(a)–4(e)). Interestingly, AP1 siRNA transfection seemed to exacerbate the increases of the TLR4 expression in these cells (Figures 4(f)–4(h)). Additionally, although AP1 siRNA transfection diminished the DNA binding activity of AP1, it seemed not be able to meliorate PA-induced increases in the CD11c mRNA expression or decreases in the CD206 mRNA expression (Figures 4(i) and 4(j)). Moreover, AP1 siRNA transfection was not able to palliate PA-induced augmentation in JNK phosphorylation (Figure 4(k)), as well as PA-induced increment in the mRNA expression or DNA binding activity of NF-*κ*B (Figures 4(l) and 4(m)). However, AP1 siRNA transfection alleviated the increases in proinflammatory factor (IL-1*β*, IL-6, and TNF-*α*) expression in RAW264.7 macrophages and TNF-*α* and IL-6 levels in the cell culture supernatant (Figures 4(n) and 4(o)). TLR4 siRNA transfection also attenuated PA-induced decreases in the mRNA expression of IL-10 (Figure 4(n)). AP1 siRNA transfection in RAW264.7 macrophages significantly moderated the decreases in AKT phosphorylation level and lipid accumulation in cocultured AML12 hepatocytes (Figures 4(p)–4(r)).

## 4. Discussion

In this study, we found that TLR4/AP-1 siRNA transfection alleviated systemic and hepatic inflammation and further ameliorated high-fat diet-induced obesity and insulin resistance in mice. Moreover, TLR4 siRNA or AP-1 siRNA transfection mitigated PA-induced inflammation in RAW264.7 cells and metabolic abnormalities in cocultured AML hepatocytes. Insulin resistance is closely related to metabolic inflammation. Macrophage polarization imbalance is a key link between obesity and lipid-induced insulin resistance in hepatocytes.

It is increasing widely considered that nutrient excess activates inflammation, causing macrophage M1 polarization and insulin resistance [3, 22]. Removal of intrahepatic macrophages in the liver tissue with chlorination improves intrahepatic inflammation independent of JNK phosphorylation and reduces circulating inflammatory factors and prevents high-fat diet-induced hepatocyte and systemic insulin resistance [8, 9], suggesting that macrophage-mediated inflammation in the liver plays an important role in the liver and systemic insulin resistance caused by high fat. Our results in this study showed that PA treatment increased the expression of proinflammatory factor (TNF-*α*, IL-1*β*, IL-6) and decreased the expression of anti-inflammatory factor (IL-10) in macrophages. The increases in TNF-*α* and IL-6 levels were also determined in the supernatant. Additionally, phosphorylation of AKT is reduced in hepatocytes noncontact cocultured with macrophages. These results suggest that PA induces macrophage production of inflammatory factors (such as TNF-*α* and IL-6) and cocultured hepatocyte insulin resistance.

In recent years, TLR4 has been recognized as an intersection of metabolism and immunity [2, 23]. It is reported that acute lipid infusion in wild-type mice is sufficient to promote adipose tissue inflammation and systemic insulin resistance, whereas these metabolic abnormalities are not observed in global TLR4-deficient mice with acute lipid infusion [23], suggesting that TLR4 plays a vital role in lipid-mediated inflammation and metabolic disorders. However, the mechanism by which TLR4 mediates inflammation and insulin resistance is probably not fully understood. In this study, we found that lentiviral-packaged TLR4-siRNA inhibited the expression of TLR4 in the liver and significantly improved metabolic inflammation and insulin resistance induced by high-fat diet. Our results indicated PA treatment significantly upregulated the expression of CD11c and CD206, proinflammatory factors, and anti-inflammatory factors and activated JNK/AP1 and IKK*β*/NF-*κ*B pathway in macrophages, as well as induced insulin resistance and lipid accumulation in hepatocytes. Moreover, TLR4 siRNA transfection alleviated PA-stimulated increases in the CD11c and CD206 expression, JNK/AP1 and IKK*β*/NF-*κ*B pathway activation, and changes of inflammatory factor levels in supernatants and herein ameliorated PA-stimulated insulin resistance and lipid deposition in cocultured hepatocytes. These results indicate that TLR4 plays an important role in the regulation of macrophage polarization in metabolic inflammation, which further lead to hepatocyte insulin resistance and lipid deposition.

JNK/AP1 is a key inflammatory pathway downstream of TLR4 [2]. It is reported that JNK plays an important role in inflammation and insulin resistance caused by high-fat diet or obesity [24, 25]. However, the role of AP1 in metabolic inflammation is far from clear. In the present study, we demonstrated that AP1 siRNA intervention attenuated PA-induced increases in proinflammatory cytokine (TNF-*α*, IL-1*β*, IL-6) gene expression in macrophages and the levels of TNF-*α* and IL-6 in cell culture supernatants. Additionally, AP1 siRNA intervention of macrophages moderated AKT phosphorylation and lipid deposition in PA-induced cocultured hepatocytes. Remarkably, AP1 siRNA intervention increased the expression of TLR4 and M1/M2 polarization markers (CD11c and CD206), implying obvious polarization imbalance of M2 to M1 transformation. These results suggest that AP1, as a key signaling molecule downstream of TLR4, did not alter the polarization state of macrophages but improved hepatocyte insulin resistance and lipid deposition by reducing the expression of proinflammatory factors.

Taken together, we propose that TLR4-AP1 signaling pathway activation plays a crucial role in high fat- or PA-induced metabolic inflammation in macrophages and insulin resistance in hepatocytes. Intervention of the TLR4 expression in macrophages regulates macrophage polarization and metabolic inflammation and herein alleviates insulin resistance and lipid deposition in hepatocytes. Downregulation of AP1 macrophages does not improve macrophage polarization but can reduce the expression of proinflammatory factors and mitigate insulin resistance and lipid deposition in hepatocytes. Therefore, TLR4 and AP1 potentially provide targets for therapeutic intervention to improve hepatic insulin resistance and lipid deposition caused by nutrient excess and lipid overload.

## Data Availability

The data used to support the findings of this study are included within the article.

## Supplementary Materials

Supplementary Materials The supplementary material provides the siRNA sequences targeting TLR4 and AP1, probe sequences used for the detection of NF-*κ*B and AP1, and the primer sequences used for real time RT-PCR.

## References

[1] Jung U. J., Choi M. S. (2014). Obesity and its metabolic complications: the role of adipokines and the relationship between obesity, inflammation, insulin resistance, dyslipidemia and nonalcoholic fatty liver disease. *International Journal of Molecular Sciences*.

[2] Li B., Leung J. C. K., Chan L. Y. Y., Yiu W. H., Tang S. C. W. (2020). A global perspective on the crosstalk between saturated fatty acids and toll-like receptor 4 in the etiology of inflammation and insulin resistance. *Progress in Lipid Research*.

[3] Petersen M. C., Shulman G. I. (2018). Mechanisms of insulin action and insulin resistance. *Physiological Reviews*.

[4] Lauterbach M. A., Wunderlich F. T. (2017). Macrophage function in obesity-induced inflammation and insulin resistance. *Pflügers Archiv - European Journal of Physiology*.

[5] Robinson M. W., Harmon C., O'Farrelly C. (2016). Liver immunology and its role in inflammation and homeostasis. *Cellular & Molecular Immunology*.

[6] Jager J., Aparicio-Vergara M., Aouadi M. (2016). Liver innate immune cells and insulin resistance: the multiple facets of Kupffer cells. *Journal of Internal Medicine*.

[7] Morinaga H., Mayoral R., Heinrichsdorff J. (2015). Characterization of distinct subpopulations of hepatic macrophages in HFD/obese mice. *Diabetes*.

[8] Papackova Z., Palenickova E., Dankova H. (2012). Kupffer cells ameliorate hepatic insulin resistance induced by high-fat diet rich in monounsaturated fatty acids: the evidence for the involvement of alternatively activated macrophages. *Nutrition & Metabolism*.

[9] Zeng T.-s., Liu F.-m., Zhou J., Pan S.-x., Xia W.-f., Chen L.-l. (2015). Depletion of Kupffer cells attenuates systemic insulin resistance, inflammation and improves liver autophagy in high-fat diet fed mice. *Endocrine Journal*.

[10] Zeng T., Zhou J., He L. (2016). Blocking nuclear factor-kappa B protects against diet-induced hepatic steatosis and insulin resistance in mice. *PLoS One*.

[11] Hu X., Zhang Q., Zhang M. (2018). Tannerella forsythia and coating color on the tongue dorsum, and fatty food liking associate with fat accumulation and insulin resistance in adult catch-up fat. *International Journal of Obesity*.

[12] Hu X., Zhang Q., Zheng J. (2017). Alteration of FXR phosphorylation and sumoylation in liver in the development of adult catch-up growth. *Experimental Biology and Medicine*.

[13] Hu X., Chen L.-L., Zheng J. (2013). Increases in systemic and local stress: a probable mechanism of visceral fat accumulation and insulin resistance in adult catch-up growth rats?. *Experimental Biology and Medicine*.

[14] Li H., Zhuang Q., Wang Y. (2014). HBV life cycle is restricted in mouse hepatocytes expressing human NTCP. *Cellular & Molecular Immunology*.

[15] Ramana K. V., Fadl A. A., Tammali R., Reddy A. B. M., Chopra A. K., Srivastava S. K. (2006). Aldose reductase mediates the lipopolysaccharide-induced release of inflammatory mediators in RAW264.7 murine macrophages. *The Journal of Biological Chemistry*.

[16] Tu F., Pang Q., Chen X., Huang T., Liu M., Zhai Q. (2017). Angiogenic effects of apigenin on endothelial cells after hypoxia-reoxygenation via the caveolin-1 pathway. *International Journal of Molecular Medicine*.

[17] Varas-Godoy M., Lladser A., Farfan N. (2018). In vivo knockdown of antisense non-coding mitochondrial RNAs by a lentiviral-encoded shRNA inhibits melanoma tumor growth and lung colonization. *Pigment Cell & Melanoma Research*.

[18] Utoyama M., Akieda-Asai S., Koda S., Nunoi H., Date Y. (2016). Role of the neural pathway from hindbrain to hypothalamus in the regulation of energy homeostasis in rats. *Neuroscience Letters*.

[19] Sinasac D. S., Riordan J. D., Spiezio S. H., Yandell B. S., Croniger C. M., Nadeau J. H. (2016). Genetic control of obesity, glucose homeostasis, dyslipidemia and fatty liver in a mouse model of diet-induced metabolic syndrome. *International Journal of Obesity*.

[20] Iwamoto M., Kurachi M., Nakashima T. (2005). Structure-activity relationship of alginate oligosaccharides in the induction of cytokine production from RAW264.7 cells. *FEBS Letters*.

[21] Park M.-J., Kim D.-I., Choi J.-H., Heo Y.-R., Park S.-H. (2015). New role of irisin in hepatocytes: the protective effect of hepatic steatosis in vitro. *Cellular Signalling*.

[22] Schenk S., Saberi M., Olefsky J. M. (2008). Insulin sensitivity: modulation by nutrients and inflammation. *The Journal of Clinical Investigation*.

[23] Shi H., Kokoeva M. V., Inouye K., Tzameli I., Yin H., Flier J. S. (2006). TLR4 links innate immunity and fatty acid-induced insulin resistance. *Journal of Clinical Investigation*.

[24] Solinas G., Vilcu C., Neels J. G. (2007). JNK1 in hematopoietically derived cells contributes to diet-induced inflammation and insulin resistance without affecting obesity. *Cell Metabolism*.

[25] Solinas G., Becattini B. (2017). JNK at the crossroad of obesity, insulin resistance, and cell stress response. *Molecular Metabolism*.

