# Supplementary material for: TLR4/AP-1-Targeted Anti-Inflammatory Intervention Attenuates Insulin Sensitivity and Liver Steatosis
Source: Mediators Inflamm. 2020 Sep 16;2020:2960517. doi: 10.1155/2020/2960517 (PMC7519185; doi:10.1155/2020/2960517)
Supplement: Supplementary Materials — The supplementary material provides the siRNA sequences targeting TLR4 and AP1, probe sequences used for the detection of NF-κB and AP1, and the primer sequences used for real time RT-PCR. [file 2960517.f1.zip › mat.2960517.v2.pdf]

Tables.

Supplemental Table 1:

A. siRNA sequences:

TLR4:	gene	F	5’- GTTTTGGCCACTGACTGAC -3’
		R	3’- GTCAGTCAGTGGCCAAAAC- 5’
	siRNA	1#	5’- GAC TTA CAG TTT CTA CGT GAA -3’
		2#	5’- GAG CTT TAG AGG TTG CTG TT -3’

		3#	5’- ACT CTA CAG ACT CCA GTT ATT -3’
AP1:	gene	F	F 5’-TGTTCATCCGTTTGTCTTCATT-3’
		R	R 5’-GCTCGCCTATTTCCTCGC-3’
	siRNA	1#	5’-CGCTCCTAAACAAACTTTGTT -3’
		2#	5’-AATGGGCACATCACCACTACA -3’
		3#	5’-AAACAGCTTCCTGCCTTTGTA -3’

B. Probe sequences:

NF-κB Probe sequences:	F	5’-AGTTGAGGGGACTTTCCCAGGC-C-(C)34-C-3’
	R	5’-GCCTGGGAAAGTCCCC-TCAACT-3’
AP1 Probe sequences:	F	5’-CGCTTGATGAGTCAGCCGGAA-C-(C)34-C-3’
	R	5’-TTCCGGCTGACTCATCAAGCG-3’

C. Primers:

Gene	Sequences	
GAPDH	Forward	5’-GGTGAAGGTCGGTGTGAACG-3’
	Reverse	5’-CTCGCTCCTGGAAGATGGTG-3’
F4/80	Forward	5’-GCTGTGAGATTGTGGAAGCA-3’
	Reverse	5’-GGCAAGACATACCAGGGAGA-3’
CD68	Forward	5’-CTTCGGGCCATGTTTCTCTT-3’
	Reverse	5’-ATTGTCGTCTGCGGGTGAT-3’
CD11c	Forward	5’-GGTGAAGGTCGGTGTGAACG-3’
	Reverse	5’-CATCAGGGAGAACCGTGTG-3’
CD206	Forward	5’-CTCTGTTCAGCTATTGGACGC-3’
	Reverse	5’-CGGAATTTCTGGGATTCAGCTTC-3’
TLR4	Forward	5’-GTTTTGGCCACTGACTGAC-3’
	Reverse	5’-GTCAGTCAGTGGCCAAAAC-3’
AP1	Forward	5’-CAGAAGAAGTTGAACGAGTA-3’

	Reverse	5’-CAGAGTCACCATTGTAGTAAT-3’
NF-κB	Forward	5’-CGGGATGGCTACTATGAG-3’
	Reverse	5’-AACCCGATTGATGAGCC-3’
IL-10	Forward	5’-CCCAACTGGTACATCAGCACCTC-3’
	Reverse	5’-GACACGGATTCCATGGTGAAGTC-3’
IL-6	Forward	5’-TAGTCCTTCCTACCCCAATTTCC-3’
	Reverse	5’-TTGGTCCTTAGCCACTCCTTC-3’
IL-1β	Forward	5’-CCCAACTGGTACATCAGCACCTC-3’
	Reverse	5’-GACACGGATTCCATGGTGAAGTC-3’
TNF-α	Forward	5’-CAGGCGGTGCCTATGTCTC-3’
	Reverse	5’-CGATCACCCCGAAGTTCAGTAG-3’
