# Supplementary material for: TLR4/AP-1-Targeted Anti-Inflammatory Intervention Attenuates Insulin Sensitivity and Liver Steatosis
Source: Mediators Inflamm. 2020 Sep 16;2020:2960517. doi: 10.1155/2020/2960517 (PMC7519185; doi:10.1155/2020/2960517)
Supplement: Supplementary Materials — The supplementary material provides the siRNA sequences targeting TLR4 and AP1, probe sequences used for the detection of NF-κB and AP1, and the primer sequences used for real time RT-PCR. [file 2960517.f1.zip › Supplementary Materials.docx]

**Tables.**

**Supplementary Materials**

The supplementary material provides the siRNA sequences targeting TLR4 and AP1, probe sequences used for the detection of NF-κB and AP1, and the primer sequences used for real time RT-PCR.

Supplemental Table 1:

1. siRNA sequences:

| TLR4: | gene | F | 5’- GTTTTGGCCACTGACTGAC -3’ |
| --- | --- | --- | --- |
|  |  | R | 3’- GTCAGTCAGTGGCCAAAAC- 5’ |
|  | siRNA | 1# | 5’- GAC TTA CAG TTT CTA CGT GAA -3’ |
|  |  | 2# | 5’- GAG CTT TAG AGG TTG CTG TT -3’ |
|  |  | 3# | 5’- ACT CTA CAG ACT CCA GTT ATT -3’ |
| AP1: | gene | F | F 5’-TGTTCATCCGTTTGTCTTCATT-3’ |
|  |  | R | R 5’-GCTCGCCTATTTCCTCGC-3’ |
|  | siRNA | 1# | 5’-CGCTCCTAAACAAACTTTGTT -3’ |
|  |  | 2# | 5’-AATGGGCACATCACCACTACA -3’ |
|  |  | 3# | 5’-AAACAGCTTCCTGCCTTTGTA -3’ |

1. Probe sequences:

| NF-κB Probe sequences: | F | 5’-AGTTGAGGGGACTTTCCCAGGC-C-(C)34-C-3’ |
| --- | --- | --- |
|  | R | 5’-GCCTGGGAAAGTCCCC-TCAACT-3’ |
| AP1 Probe sequences: | F | 5’-CGCTTGATGAGTCAGCCGGAA-C-(C)34-C-3’ |
|  | R | 5’-TTCCGGCTGACTCATCAAGCG-3’ |

1. Primers:

| Gene | Sequences | |
| --- | --- | --- |
| GAPDH | Forward | 5’-GGTGAAGGTCGGTGTGAACG-3’ |
|  | Reverse | 5’-CTCGCTCCTGGAAGATGGTG-3’ |
| F4/80 | Forward | 5’-GCTGTGAGATTGTGGAAGCA-3’ |
|  | Reverse | 5’-GGCAAGACATACCAGGGAGA-3’ |
| CD68 | Forward | 5’-CTTCGGGCCATGTTTCTCTT-3’ |
|  | Reverse | 5’-ATTGTCGTCTGCGGGTGAT-3’ |
| CD11c | Forward | 5’-GGTGAAGGTCGGTGTGAACG-3’ |
|  | Reverse | 5’-CATCAGGGAGAACCGTGTG-3’ |
| CD206 | Forward | 5’-CTCTGTTCAGCTATTGGACGC-3’ |
|  | Reverse | 5’-CGGAATTTCTGGGATTCAGCTTC-3’ |
| TLR4 | Forward | 5’-GTTTTGGCCACTGACTGAC-3’ |
|  | Reverse | 5’-GTCAGTCAGTGGCCAAAAC-3’ |
| AP1 | Forward | 5’-CAGAAGAAGTTGAACGAGTA-3’ |
|  | Reverse | 5’-CAGAGTCACCATTGTAGTAAT-3’ |
| NF-κB | Forward | 5’-CGGGATGGCTACTATGAG-3’ |
|  | Reverse | 5’-AACCCGATTGATGAGCC-3’ |
| IL-10 | Forward | 5’-CCCAACTGGTACATCAGCACCTC-3’ |
|  | Reverse | 5’-GACACGGATTCCATGGTGAAGTC-3’ |
| IL-6 | Forward | 5’-TAGTCCTTCCTACCCCAATTTCC-3’ |
|  | Reverse | 5’-TTGGTCCTTAGCCACTCCTTC-3’ |
| IL-1β | Forward | 5’-CCCAACTGGTACATCAGCACCTC-3’ |
|  | Reverse | 5’-GACACGGATTCCATGGTGAAGTC-3’ |
| TNF-α | Forward | 5’-CAGGCGGTGCCTATGTCTC-3’ |
|  | Reverse | 5’-CGATCACCCCGAAGTTCAGTAG-3’ |
